# Choroidal thickness in Chinese patients with non-arteritic anterior ischemic optic neuropathy

**DOI:** 10.1186/s12886-016-0313-2

**Published:** 2016-08-31

**Authors:** Libin Jiang, Lanlan Chen, Xiujuan Qiu, Ran Jiang, Yaxing Wang, Liang Xu, Timothy Y. Y. Lai

**Affiliations:** 1 Beijing Tongren Eye Center, Beijing Tongren Hospital, Capital Medical University, Beijing Ophthalmology and Visual Sciences Key Laboratory, Beijing, China; 2 Department of Ophthalmology, Hainan Branch of Chinese People’s Liberation Army General Hospital, Sanya, Hainan China; 3 Beijing Institute of Ophthalmology, Beijing Tongren Eye Center, Beijing Tongren Hospital, Capital Medical University, Beijing, China; 4 Department of Ophthalmology & Visual Sciences, The Chinese University of Hong Kong, Hong Kong Eye Hospital, Kowloon, Hong Kong, China

**Keywords:** Non-arteritic anterior ischemic optic neuropathy, Choroidal thickness, Optical coherence tomography

## Abstract

**Background:**

Non-arteritic anterior ischemic optic neuropathy (NA-AION) is one of the most common types of ischemic optic neuropathy. Several recent studies suggested that abnormalities of choroidal thickness might be associated with NA-AION. The main objective of this case–control study was to evaluate whether choroidal thickness is an ocular risk factor for the development of NA-AION by evaluating the peripapillary and subfoveal choroidal thicknesses in affected Chinese patients.

**Methods:**

Forty-four Chinese patients with unilateral NA-AION were recruited and compared with 60 eyes of 60 normal age and refractive-error matched control subjects. Peripapillary and subfoveal choroidal thicknesses were measured by enhanced depth imaging optical coherence tomography. Choroidal thicknesses of eyes with NA-AION and unaffected fellow eyes were compared with normal controls. Choroidal thicknesses of NA-AION eyes with or without optic disc edema were also compared. The correlation between choroidal thickness and retinal nerve fiber layer (RNFL) thickness, logMAR best-corrected visual acuity (BCVA), and the mean deviation (MD) of Humphrey static perimetry in NA-AION eyes were analyzed.

**Results:**

The peripapillary choroidal thicknesses at the nasal, nasal inferior and temporal inferior segments in NA-AION eyes with optic disc edema were significantly thicker compared with that of normal subjects (*P* < 0.05). There was no significant difference in the choroidal thicknesses between the unaffected fellow eyes of NA-AION patients and normal eyes of healthy controls; or between the NA-AION eyes with resolved optic disc edema and normal eyes (all *P* > 0.05). No significant correlation between choroidal thickness and RNFL thickness, logMAR BCVA and perimetry MD was found in eyes affected by NA-AION (all *P* > 0.05).

**Conclusions:**

Increase in peripapillary choroid thickness in some segments was found in NA-ION eyes with optic disc edema. However, our findings do not support the hypothesis that choroidal thickness is abnormal in Chinese patients with NA-AION compared with normal subjects with similar age and refractive error status.

## Background

Non-arteritic anterior ischemic optic neuropathy (NA-AION) is one of the most common types of ischemic optic neuropathy and can develop bilaterally over an interval of several months to years. The development of NA-AION is caused by acute ischemia of the optic nerve head (ONH), in which the main source of blood supply is derived from the posterior ciliary artery circulation via the peripapillary choroid. Therefore, it has been suggested that the peripapillary choroidal thickness might be altered following ONH ischemic disorders [1]. In addition, it has been shown that an absent or small cup in a small optic disc is the most important ONH risk factors for development of NA-AION [1]. Abnormalities of choroidal thickness have been found to be associated with various ocular diseases including glaucoma and various maculopathies [2–6]. However, it remains uncertain whether abnormal choroidal thickness associated with small optic disc will increase the risk of NA-AION development. Schuster et al. [7] investigated the subfoveal choroidal thickness (SFCT) in patients with NA-AION and they found that the unaffected contralateral fellow eyes of NA-AION patients was significantly thinner compared with eyes of a control group after adjusting various ocular and systemic parameters. In contrast, Fard et al. [8] recently found that peripapillary choroidal thickness in Iranian NA-AION patients was thicker than control eyes. It is unknown whether choroidal thickness in Chinese NA-AION patients is normal or not. The aim of our study was to measure the peripapillary and subfoveal choroidal thicknesses in Chinese patients with NA-AION and compare with normal eyes of healthy subjects in order to assess whether choroidal thickness is another ocular risk factor for the development of NA-AION.

## Methods

This observational case–control study consisted of 44 Chinese patients with unilateral NA-AION and 60 healthy Chinese volunteers. The participants were examined between October 2012 and June 2014. The study adhered to the tenets of the Declaration of Helsinki and was approved by the Institutional Review Board and Ethics Committee of Capital Medical University and informed consent were obtained from all participants. The reporting of the study adhered to the STROBE guidelines for case–control studies. The diagnostic criteria for NA-AION included a history of sudden visual loss; optic disc edema at the disease onset; spontaneous resolution of optic disc edema at subsequent follow-up; optic disc-related visual field defects; and an absence of other ocular, systemic, or neurologic diseases that might influence the patient’s visual symptoms or cause optic disc edema and visual impairment [1,9]. Exclusion criteria included any history of eye diseases or any kind of intraocular surgery or laser photocoagulation, and history corticosteroids, sildenafil or related drugs use. Sample size was based on the number of patients presented to the clinic with NA-AION. Patients with NA-AION were further divided into two groups with or without optic disc edema.

**Fig 1:**
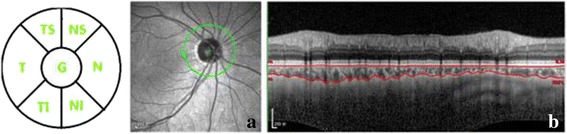
Enhanced depth optical coherence tomography scan with 360° 3.4 mm diameter peripapillary circle scan for measurement of choroidal thickness. **a** Diagram showing segmentation of the peripapillary choroidal thickness measurements in the arithmetic mean of sum in each segment (G) and six segments including nasal superior (NS), nasal (N), nasal inferior (NI), temporal inferior (TI), temporal (T), temporal superior (TS) segments. **b** Manual delineation of the choroidal layer between the outer border of the retinal pigment epithelium and the inner surface of the sclera for measurement of choroidal thickness

All participants underwent a thorough ophthalmic examination, which included measurement of logMAR best-corrected visual acuity (BCVA), refraction, tonometry, slit-lamp biomicroscopy, fundus examination, axial length measurements using A-scan ultrasonography, and 24–2 Humphrey automated static perimetry. Peripapillary and subfoveal choroidal thicknesses were measured through natural pupil size using the enhanced depth imaging mode of the Heidelberg Spectralis optical coherence tomography (EDI-OCT, Heidelberg engineering, Software Version 5.3.2). Subfoveal choroidal thickness was measured by a horizontal scan going directly through the center of the fovea and the peripapillary choroid thickness was measured by a 360°, 3.4 mm diameter peripapillary circular scan using the standard protocol for retinal nerve fiber layer (RNFL) as previously described [6,10–12]. Choroidal thickness was defined as the perpendicular vertical distance from the hyper-reflective line of Bruch’s membrane to the hyper-reflective line of the inner surface of sclera [6,10–12]. Peripapillary choroidal thickness was calculated by the arithmetic mean of each segment (G) and six individual segments including the nasal superior (NS), the nasal (N), the nasal inferior (NI), the temporal inferior (TI), the temporal (T), the temporal superior (TS) (Fig. 1). All OCT scans and measurements were performed by two masked examiners independently. If the measurements of the two examiners differed by more than 15 %, the examiners were asked to repeat the measurement together to reach a consensus.

Statistical analysis was performed using a commercially available statistical software package (SPSS for Windows, version 19.0; IBM-SPSS, Chicago, IL). We used independent sample t-test to analyze factors related with choroidal thickness between NA-AION patients and normal subjects including age, gender, refractive error and axial length. Secondly, we performed t-test to compare the choroidal thickness in the affected eyes of NA-AION patients with normal eyes of healthy control subjects, and the unaffected fellow eyes of NA-AION patients with eyes of controls. Further analysis was performed to compare eyes with or without optic disc edema and normal eyes of healthy subjects. Bivariate correlation analysis was performed to evaluate the relationships between choroidal thickness and RNFL thickness, logMAR BCVA, and the mean deviation (MD) of Humphrey static perimetry (24–2) of the affected eyes of NA-AION patients. *P* values were two-sided and were considered statistically significant when the values were <0.05.

## Results

### Patients’ demographics

A total of 44 Chinese patients (23 males and 21 females) with unilateral NA-AION were included in the study. None of the patients was excluded due to failure to fulfill the exclusion or inclusion criteria. The mean ± standard deviation (SD) age of the patients was 50.8 ± 10.0 years (range, 35 to 66 years) and the mean ± SD axial length was 22.69 ± 0.88 mm (range, 21.23 to 23.44 mm). The mean ± SD spherical equivalent refractive error of the eyes with NA-AION was 0.78 ± 1.14D (range: +1.00D to −3.00D). The baseline characteristics of the NA-AION patients are shown in Table 1. The normal control group consisted of 60 eyes of 60 healthy subjects (30 males and 30 females) with a mean ± SD age of 50.3 ± 1.2 years, mean ± SD axial length of 22.83 ± 0.78 mm and mean spherical equivalent refractive error of −0.18 ± 1.98D. There was no significant difference in the baseline characteristics including mean age, axial length and refractive error between the NA-AION patients with or without optic disc edema and controls (all *P* > 0.05). Therefore, we compared choroidal thickness between patients in NA-AION and normal control group without adjusting for age, axial length and refractive error.

**Table 1:** Baseline characteristics including age, axial length and refractive error in NA-AION patients with and without optic disc edema

	Female/male (n)	Mean age (mean ± SD years)	Mean axial length (mean ± SD mm)	Spherical equivalent refractive error (mean ± SD D)
NA-AION patients with optic disc edema (*n* = 19)	9/10	50.63 ± 10.92	22.57 ± 0.62	0.52 ± 0.54
NA-AION patients without optic disc edema (*n* = 25)	12/13	51.00 ± 9.37	22.57 ± 1.14	0.54 ± 1.63

### Choroidal thickness of the eyes affected by NA-AION and the unaffected fellow eyes compared with normal subjects

Peripapillary choroidal thicknesses in different segments and subfoveal choroidal thicknesses of the affected and fellow eyes of NA-AION patients and that of normal subjects are shown in Table 2. Peripapillary choroid in the nasal, nasal inferior and temporal inferior segments in the eyes affected by NA-AION were thicker than normal subjects with similar age and refractive error status (*P*<0.05). However, we found no difference between subfoveal choroidal thickness of the NA-AION affected eyes and that of normal subjects; nor between the peripapillary and subfoveal choroidal thickness of the unaffected fellow eyes of NA-AION patients and that of normal subjects (all *P* > 0.05).

**Table 2:** Peripapillary choroidal thickness in different segments and subfoveal choroidal thickness in NA-AION patients and normal subjects (mean ± SD) μm

	TS	NS	N	NI	TI	T	G	SFCT
Normal control group (*n* = 60)	187.72 ± 49.12	178.90 ± 47.89	163.42 ± 42.27	148.60 ± 43.75	148.28 ± 45.05	180.03 ± 55.50	169.30 ± 43.04	296.40 ± 68.36
Affected eyes (*n* = 44)	197.98 ± 82.11	199.23 ± 75.78	193.27 ± 70.79	179.48 ± 85.62	174.16 ± 71.55	199.89 ± 87.37	191.55 ± 73.58	305.79 ± 105.93
*P* value	0.46	0.12	0.02	0.03	0.03	0.19	0.07	0.64
Fellow eyes (*n* = 44)	196.98 ± 65.66	193.64 ± 66.26	183.64 ± 75.14	159.93 ± 69.62	162.98 ± 71.25	195.11 ± 81.67	183.77 ± 69.32	311.46 ± 83.89
*P* value	0.41	0.19	0.11	0.35	0.23	0.29	0.23	0.39

Note: TS, NS, N, NI, TI and T represented respectively peripapillary choroidal thickness in the temporal superior, nasal superior, nasal, nasal inferior, temporal inferior, and temporal segments, G the mean peripapillary choroidal thickness, SFCT subfoveal choroidal thickness

### Choroidal thickness in NA-AION eyes with or without optic disc edema compared with normal eyes

The subfoveal and peripapillary choroidal thicknesses in various segments in the affected and fellow eyes of NA-AION patients with or without optic disc edema are shown in Table 3. Peripapillary choroid in the nasal, nasal inferior and temporal inferior segments in eyes affected by NA-AION with optic disc edema were thicker than normal subjects with similar age and refractive error (*P*<0.05). However, we found no significant difference between the subfoveal choroidal thickness of NA-AION eyes with optic disc edema and that of normal subjects; and between the peripapillary and subfoveal choroidal thicknesses of the affected eyes of NA-AION patients without optic disc edema and that of normal subjects (all *P* > 0.05).

**Table 3:** Peripapillary choroidal thickness in different segments and subfoveal choroidal thickness of the NA-AION patients with or without optic disc edema (mean ± SD) μm

	TS	NS	N	NI	TI	T	G	SFCT
Normal control group (*n* = 60)	187.72 ± 49.12	178.90 ± 47.89	163.42 ± 42.27	148.60 ± 43.75	148.28 ± 45.05	180.03 ± 55.50	169.30 ± 43.04	296.40 ± 68.36
NA-AION eyes with optic edema (*n* = 19)	202.63 ± 78.38	208.05 ± 70.70	204.42 ± 67.75	200.31 ± 100.95	188.26 ± 76.57	214.00 ± 87.87	203.26 ± 72.29	313.56 ± 82.49
*P* value	0.44	0.11	0.02	0.04	0.04	0.13	0.07	0.42
NA-AION eyes without optic disc edema (*n* = 25)	194.44 ± 86.27	192.52 ± 80.20	184.80 ± 73.22	163.64 ± 69.91	163.44 ± 67.06	189.16 ± 87.22	182.64 ± 74.76	300.13 ± 121.79
*P* value	0.72	0.43	0.18	0.33	0.31	0.63	0.41	0.90

Note: TS, NS, N, NI, TI and T represented respectively peripapillary choroidal thickness in the temporal superior, nasal superior, nasal, nasal inferior, temporal inferior, and temporal segments, G the mean peripapillary choroidal thickness, SFCT subfoveal choroidal thickness

### Correlation between choroidal thicknesses and RNFL thickness, BCVA, and visual field MD

Bivariate correlation analysis was performed to evaluate the association between various parameters and peripapillary and subfoveal choroidal thicknesses. No significant correlation was found between choroidal thickness and peripapillary RNFL thickness, logMAR BCVA, and MD of Humphrey visual field in eyes with NA-AION (all *P* > 0.05). In addition, no significant correlation was found between the peripapillary choroidal thicknesses in each segment and subfoveal choroidal thicknesses in both the affected and fellow eyes of NA-AION patients (all *P* > 0.05).

## Discussion

Previous studies on choroidal thickness in various retinal, chorioretinal and optic nerve head disorders have revealed that SFCT was reduced with older age and increasing myopic refractive error, and SFCT was reduced in myopic maculopathy and in eyes with geographic atrophy [5]. Moreover, it has been found that the choroidal thickness in the macular region in eyes of acute primary angle-closure (APAC) patients was greater compared with eyes of primary angle-closure suspect [6]. The unaffected fellow eyes of patients in which one eye previously developed APAC were also found to have thicker macular choroid [6]. However, in eyes with normal-tension glaucoma (NTG), the mean peripapillary choroidal thickness was thinner than normal subjects [13]. In NA-AION, Schuster et al [7] reported that the SFCT of both the affected and unaffected contralateral eyes in NA-AION patients were thinner than control eyes after adjusting ocular and systemic parameters. However, since the study was a cross-sectional study, it could not be concluded that thin choroid is a risk factor for NA-AION. Several ocular risk factors including absent or small optic disc cup and angle closure glaucoma have been shown to be associated with NA-AION. Some studies have also demonstrated that patients with NA-AION were more hypermetropic compared with age-matched control group [14,15]. These findings suggested that the choroidal thickness in patients with NA-AION should not be thinner than that in an age-matched control group. Recently, Fard et al [8] evaluated the peripapillary choroidal thickness in Iranian patients with NA-AION using spectral-domain OCT and found that the peripapillary choroidal thickness was thicker in both the affected and unaffected eyes of NA-AION patients compared with control eyes. In our study, the peripapillary and subfoveal choroidal thicknesses of Chinese patients with NA-AION were measured using EDI-OCT. As no significant difference was found in the mean age, refractive error and axial length between the NA-AION patients and the control group, we evaluated the peripapillary and subfoveal choroidal thicknesses of NA-AION eyes and compared with normal eyes without adjusting for age, axial length and refractive error. In contrast to the findings by Schuster et al. [7] and Fard et al. [8], our results demonstrated that the peripapillary choroid in the nasal, nasal inferior and temporal inferior segments of eyes with NA-AION eyes were thicker than normal subjects. Moreover, we did not find a significant difference either between SFCT of eyes with NA-AION and that of normal subjects, and between the peripapillary and subfoveal choroidal thicknesses of the unaffected fellow eyes of the NA-AION patients and that of normal subjects.

In order to evaluate the impact of optic disc edema on the peripapillary choroidal thicknesses, we measured the choroidal thicknesses of NA-AION eyes with optic disc edema and those with resolved optic disc edema and found that the peripapillary choroidal thicknesses in the nasal, nasal inferior and temporal inferior segments in eyes with optic disc edema were thicker than normal subjects. We found no significant difference between the choroidal thickness in eyes with resolved optic disc edema and that in normal subjects, nor between the choroidal thickness of the unaffected fellow eyes of the NA-AION patients and that of normal subjects. The results suggest that abnormal thickening of the peripapillary choroid in some segments may be pathologically attributed to optic disc edema in early stage of NA-AION, rather than contributing to the development of NA-AION. Alterations in the peripapillary choroid observed in eyes with NA-AION are therefore likely to be secondary effects rather than the cause of NA-AION. The reason for the thickening of peripapillary choroid in the nasal, nasal inferior and temporal inferior segments might be related to expansion of peripapillary choroidal blood capillaries. The combination of a relative inferior altitudinal defect with an absolute inferior nasal defect is the most common pattern of visual field defect in NA-AION, suggesting the presence of vascular insufficiency involving the temporal and superior parts of the capillary bed of the ONH, and a vascular compensatory expansion in the nasal and inferior parts of the capillary bed at early stage of NA-AION [1]. As the peripapillary choroidal vasculature and the ONH vasculature are closely connected to each other, expansion of the ONH vasculature causes increase in peripapillary choroidal blood flow. Following resolution of optic disc edema in the recovery stage of NA-AION, the vascular compensatory expansion disappears and the peripapillary choroid thickness returns back to normal. The peripapillary choroid thickness might even be thinner with the development of optic nerve atrophy.

Studies reported in the literature to date have shown no consensus on the correlation between RNFL and choroidal thicknesses. Zhao et al [16] believed that thicker RNFL thickness was associated with thicker SFCT, while Suh et al [17] suggested that there was no correlation between RNFL and choroidal thickness in unilateral NTG. In the Beijing Eye Study, Shao et al [18] reported that better BCVA was strongly associated with thicker subfoveal choroid independent of other factors such as age, axial length, education level, and ocular diseases including glaucoma, diabetic retinopathy and late-stage age-related macular degeneration. Our results showed no significant correlation between choroidal thicknesses and BCVA, visual field MD or RNFL thickness in the affected eyes of the NA-AION patients.

There were some limitations in our study. Firstly, our study was a cross-sectional study and we were unable to perform serial EDI-OCT measurements in NA-AION patients. Therefore, the group with optic disc edema and the group without optic disc edema were not self-matching and we lacked data about the longitudinal follow-up of the choroidal thicknesses in the patients with optic disc edema. Secondly, most of the NA-AION patients suffered from one or more systemic diseases such as hypertension, hyperlipidemia and diabetes mellitus. Since SFCT was found not to be significantly associated with blood pressure, serum concentrations of lipids, and serum glucose [19], we compared the peripapillary choroidal thickness of eyes with NA-AION with that of a normal control group without adjusting for blood pressure and serum concentrations of lipids and glucose. These might potentially influence the results of our study.

## Conclusions

The present study showed that increase in peripapillary choroidal thickness occurred in some peripapillary choroid segments in NA-AION eyes with optic disc edema and not in NA-AION eyes without optic disc edema compared with eyes of normal subjects with similar age, refractive error status and axial length. The findings showed there is insufficient evidence to demonstrate that choroidal thickness is abnormal in eyes with NA-AION compared with normal subjects, suggesting that choroidal thickness is unlikely to be an ocular risk factor for the development of NA-AION.

## Abbreviations

APAC, acute primary angle-closure; BCVA, best-corrected visual acuity; EDI-OCT, enhanced depth imaging-optical coherence tomography; MD, mean deviation; NA-AION, non-arteritic anterior ischemic optic neuropathy; NTG, normotension glaucoma; ONH, optic nerve head; RNFL, retinal nerve fiber layer; SFCT, subfoveal choroidal thickness
